# Comparison of movement related cortical potential in healthy people and amyotrophic lateral sclerosis patients

**DOI:** 10.3389/fnins.2013.00065

**Published:** 2013-05-14

**Authors:** Ying Gu, Dario Farina, Ander R. Murguialday, Kim Dremstrup, Niels Birbaumer

**Affiliations:** ^1^Center for Sensory-Motor Interaction, Aalborg UniversityAalborg, Denmark; ^2^Department of Neurorehabilitation Engineering, Georg-August UniversityGöttingen, Germany; ^3^Institute of Medical Psychology and Behavioral Neurobiology, Eberhard-Karls-UniversityTübingen, Germany; ^4^TECNALIA, Health TechnologiesSan Sebastian, Spain; ^5^Ospedale San Camillo, IRCCSVenice, Italy

**Keywords:** electroencephalography (EEG), movement related cortical potential (MRCP), movement imagination, brain computer interface (BCI), amyotrophic lateral sclerosis (ALS)

## Abstract

**Objective:** To understand the brain motor functions and neurophysiological changes due to motor disorder by comparing electroencephalographic data between healthy people and amyotrophic lateral sclerosis (ALS) patients.

**Methods:** The movement related cortical potential (MRCP) was recorded from seven healthy subjects and four ALS patients. They were asked to imagine right wrist extension at two speeds (fast and slow). The peak negativity (PN) and rebound rate (RR) were extracted from MRCP for comparison.

**Results:** The statistical analysis has showed that there was no significant difference in PN between the healthy and the ALS subjects. However, the healthy subjects presented faster RR than ALS during both fast and slow movement imagination.

**Conclusions:** The weaker RR of ALS patients might reflect the impairment of motor output pathways or the degree of motor degeneration.

**Significance:** The comparison between healthy people and ALS patients provides a way to explain the movement disorder through brain electrical signal. In addition, the characteristics of MRCP could be used to monitor and guide brain plasticity in patients.

## Introduction

Movement Related Cortical Potential (MRCP) represents the electroencephalographic (EEG) evidence of motor cortical involvement during movement and movement preparation (Kornhuber and Deecke, 1965). The MRCP belongs to the family of slow cortical potentials (SCPs) which reflect the summed dendritic postsynaptic potentials of cortical pyramidal neurons arranged perpendicular to the cortical surface (Birbaumer et al., 1990; Niedermeyer and Lopes da Silva, 1999). It is detected usually by averaging repeated EEG epochs in the time domain. The MRCP consists of a pre-movement potential also called Bereitschaftspotential (BP) and a post-movement potential. The BP consists of several independent components with different cortical sources and different physiological functions. A concise description of the physiological and behavioral meaning of the different components of the family of SCP can be found in Rockstroh et al. (1989). The post-movement potential is believed to reflect the reafferent feedback and fine control of a movement (Jahanshahi and Hallett, 2003; do Nascimento et al., 2005). Different terminologies have been proposed for identifiable pre-movement components and post-movement components according to the spatial and temporal distribution of the components (Jahanshahi and Hallett, 2003). In this study we extracted Peak Negativity (PN) (the maximal point of BP) and positive Rebound Rate (RR) of post-movement potential for analysis.

The MRCP has been studied for decades mostly in motor control physiology and psychophysiology (Shibasaki et al., 1981; Libet et al., 1983a,b; Slobounov et al., 1998; Jahanshahi and Hallett, 2003). It is known that MRCP occurs in association with both executed and imagined movements and that its magnitude and latency are modulated by the participants' psychological status and the characteristics of the movement performed, such as speed, precision, and movement repetition (Birbaumer et al., 1990; Slobounov et al., 1998; Romero et al., 2000; do Nascimento et al., 2005, 2006; Nielsen et al., 2006). The modulation of MRCP, especially with motor imagery, leads to an important perspective in rehabilitation technology. Recently, efforts have been devoted to identify MRCP in single trial basis for their application in Brain computer interface (BCI) (Farina et al., 2007; do Nascimento and Farina, 2008; Gu et al., 2009a,b,c).

BCI aims to provide a no-muscular communication and control channel for severely disabled patients (Birbaumer et al., 1999; Wolpaw et al., 2002). In addition, BCI might contribute to neurological rehabilitation by guiding and facilitating brain plasticity (Daly and Wolpaw, 2008; Wang et al., 2010). MRCP can be one of suitable signals for monitoring and guiding brain plasticity for motor restoration (Jahanshahi and Hallett, 2003; Shibasaki and Hallett, 2006).

Amyotrophic Lateral Sclerosis (ALS) leads to severe motor disorders and paralysis. Specifically in ALS, the disease progresses from the first symptoms of muscular or respiratory weakness to the locked in (LIS) and the complete locked in state (CLIS). In these patients, sensory, emotional, and cognitive processing often remains largely intact despite extensive degeneration of the motor system (Kübler et al., 2005) at least until the CLIS state (Ramos Murguialday et al., 2011). Modern life support technology allows longer life expectancies and therefore surviving patients with neurodegenerative diseases are and will be more frequent in the future. Motor impairment greatly limits independent living and social interaction which are responsible for a good quality of life (Kübler et al., 2007). BCI is a possible solution for those patients affected by motor disabilities supporting and assisting in the interaction with the environment. The first report of a BCI-based system in advanced ALS with (LIS) used SCP as the critical output (Birbaumer et al., 1999). However, long periods of training already pointed towards a pathophysiological modification of the SCP. BCI based SCP has been tested extensively in late-stage ALS and has proven able to supply basic communication capabilities (Kübler et al., 2007; Birbaumer et al., 2008). It has been reported that by control of SensoriMotor Rhythm (SMR) amplitude, patients with LIS can spell using so-called virtual keyboard (Obermaier et al., 2003). The advantage of using MRCP in BCI system is that the control may require much less training time and it could serve as an alternative or supplementary control signal.

In this study, we analyzed the characteristics of MRCP in ALS and compared it with that from healthy volunteers. The comparison is expected to contribute to a better understanding of brain processes of motor functions and help us understand the neurophysiological changes due to the motor disorder. Moreover, it might help to transfer BCI technology based on healthy people's data to BCI systems for ALS patients. We conducted two different motor imagery experiments with comparable experimental set up, protocol, and signal analysis. The aim of this study was to describe the differences between healthy people and ALS MRCPs in order to prepare the BCI community for possible modification in the technology for brain-based communication in ALS.

## Materials and methods

### Participants

Seven healthy volunteers (3 men and 4 women) aged 25–30 years and 4 ALS patients (1 man and 3 women) aged 40–70 years were involved in the study. None of the healthy volunteers reported any sensory-motor diseases or any clinical history of psychological disorders. The ALS patients were evaluated and given ALS Functional Rating Scale (ALS-FRS) score before study. ALS-FRS is a score of 0–40 which assess the severity of ALS patient (Cefarbaum and Stambler, 1997). The higher score, the more functions are retained. The detailed information of ALS patients is described in Table 1. The experiment protocol was approved by the local ethics committee in Aalborg (healthy subjects) and the ethics committee of the Medical Faculty of the University of Tübingen (patients). The informed consent was obtained from all subjects.

**Table 1:** **ALS patients' characteristics**.

**Patient no.**	**Age**	**Gender**	**Degree of physical impairment**	**Speech**	**ALS-FRS**
1	40	Female	No movement of upper limbs; very limited lower limbs' movement; unstable eye control	Impaired	7
2	46	Female	No movement of right wrist; other limbs' movement limited; normal eye control	Normal	14
3	51	Female	Locked in state, artificially fed, and ventilated; unstable eye control	No speech	1
4	70	Male	Intact limbs' movement, except for a slight weakness on the right index finger; normal eye control	Normal	38

ALS-FRS: 0, worst; 40, best.

### Experimental procedure

The participants were seated on a comfortable chair and were asked to imagine right wrist extension at two speeds (fast and slow). The fast speed corresponded to a movement executed as fast as possible whereas slow speed was associated to a movement performed in approximately 3 s. The tasks were randomly presented to the participants, controlled by a computer program developed by LabVIEW 8.2.1. The EEG/EOG (electrooculographic) signals were amplified with a digital DC EEG amplifier (Neuro Scan Labs, NuAmps), low-pass filtered with cut-off frequency 200 Hz and sampled at 500 Hz using a 22-bit A/D converter. The EEG (F3, Fz, F4, C3, Cz, C4, P3, Pz, and P4) was recorded with software Scan43 (Neuro Scan Labs). The electrodes' impedances were kept below 5 kΩ. For healthy subjects, surface electromyographic (EMG) signals were recorded from the extensor carpi ulnaris and palmaris longus muscles using self-adhesive disposable electrodes. The EMG was used to monitor unwanted wrist movement during the imagined movement. The participants were asked to avoid eye blinking, slow eye movement, and facial movement during motor imagery. We conducted two separate experiments as follows:

- EEG recordings from 7 healthy volunteers: The word for the movement (“fast” or “slow”) to be imagined was displayed on the screen. After a random time interval of 2~3 s, a visual cue indicated the onset of the imagination task. The subject familiarized by executing the tasks by approximately 3 min. They were instructed to perform kinesthetic imagery while avoiding any overt muscle activity during recording session.
- EEG recordings from 4 ALS patients: Since two patients could not reliably control their gazes, an auditory cue consisting of a voice recording pronouncing the name of the specific movement, indicated the required action for each trial. After a random time interval of 2~3 s, a “beep” indicated the beginning of the imagery task. The experimenter instructed the patients how to perform the imagery. In addition, the experimenter passively moved the patients' right wrist to enhance the kinesthetic sensation of the imagery to be performed during the instruction session. The subjects were asked to feel themselves moving instead of merely visualizing the limb movement. During recording session, the subjects only performed movement imagination.

### MRCP and statistical analysis

Epochs starting 2 s before the imagination onset and 2 s after were extracted using EEGlab software (Delorme and Makeig, 2004). Trials identified visually as contaminated by EOG signals exceeding 75 uV were rejected from further analysis. Further trials contaminated by facial EMG which appeared in the EEG recording channels were discarded. The baseline was corrected on each EEG channel by subtracting the mean amplitude value in the interval −2 s to −1.8 s referenced to the imagination onset (time 0).

The PN and RR were identified in a single trial basis. Firstly, the EEG signals were smoothed using a moving average over 400 time samples (Smith, 2003). Then, the PN was calculated as the lowest value between −1 and 2 s. The RR was calculated as: $\text{RR}=\frac{\text{MRCP}(T+t)-\text{MRCP}(T)}{t}$.

where *T* was the time point of PN and *t* was the time interval over which RR was computed. *t* was chosen as 1 s empirically in this study.

Finally, the averaged PN and RR were calculated for each subject for further statistical analysis. One-sided wilcoxon rank-sum test, a non-parametric statistical significance test, was performed to test for significant differences on PN and RR between healthy subjects and ALS patients. Outcomes were considered significant if *p* < 0.05.

## Results

**Figure 1:**
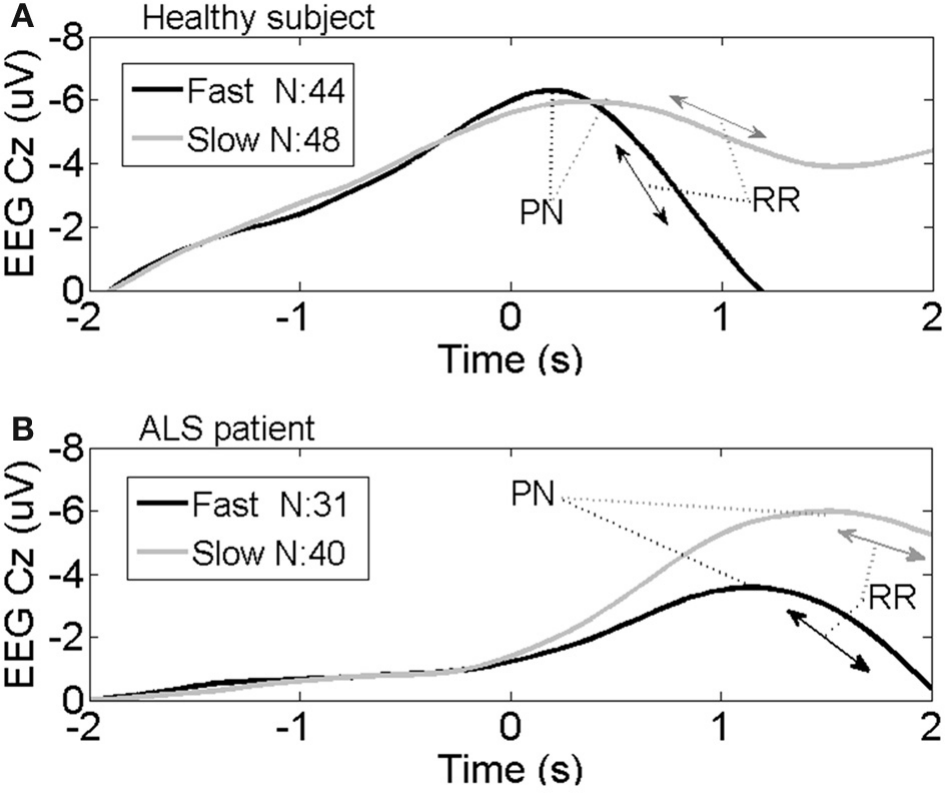
**Average MRCP from one representative healthy subject (A) and one representative ALS patient (B) at channel Cz during the fast and slow speed tasks.** Imaginary movement onset is represented by time 0 s; *N*, number of averaged trials; PN, Peak Negativity; RR, Rebound Rate.

Figures 1A,B show averaged MRCP from one representative healthy subject and one ALS patient, respectively. These two plots show the typical time course of MRCP. The negativity started to rise around –2 s. After the imagery onset (time 0), the potential reached its maximum negativity. RRs between fast and slow speed were quite different in the healthy volunteer, although the differences in BP and PN between fast and slow were not significant by visual inspection (see Figure 1A). In Figure 1B, there was a difference in PN latency between slow and fast movement imagery in the ALS patient. Visually in Figures 1A,B, RRs were different between the representative healthy subject and ALS patient.

Table 2 shows the averages and standard deviations for PN and RR at the two speeds for each healthy subject and ALS patient. One-sided wilcoxon rank-sum test has been performed between 7 healthy subjects and ALS patient 1, 2, and 3 on Fast PN, Slow PN, Fast RR, and Slow RR, separately. ALS patient 4 was excluded from the patient group due to the fact that he was in very early stage of disease and had normal wrist movements, while other three ALS patients had no right wrist movement at all. The statistical analysis shows that there were significant differences on Fast RR (*p* = 0.008) and Slow RR (*p* = 0.017) between the healthy and ALS patients. However, there was no significant difference on Fast PN and Slow PN between the healthy and ALS. Healthy subjects showed faster RR than ALS during both fast and slow speed task. Patient 4 showed quite similar RR as the healthy.

**Table 2:** **Average PN, RR at Cz for each healthy subject and ALS patient**.

	**Number of trials Fast/Slow**	**Fast PN (μV)**	**Slow PN (μV)**	**Fast RR (μV)**	**Slow RR (μV)**
**HEALTHY SUBJECTS**					
1	44/48	−7.39	−8.69	7.54	6.65
2	35/29	−9.73	−17.22	15.56	12.79
3	29/28	−18.45	−30.78	14.81	13.18
4	34/33	−24.96	−20.76	18.54	12.56
5	27/35	−11.03	−11.04	9.40	7.76
6	48/42	−9.20	−8.20	8.88	9.97
7	37/36	−10.00	−13.44	7.87	4.97
	Mean ± *SD*	−12.97 ± 6.35	−15.73 ± 8.04	11.80 ± 4.41	9.70 ± 3.30
**ALS PATIENTS**					
1	35/38	−12.51	−11.37	4.34	5.11
2	31/40	−7.04	−7.92	4.75	3.62
3	36/38	−13.31	−11.71	5.60	4.27
	Mean ± *SD*	−10.95 ± 3.51	−10.33 ± 2.10	4.90 ± 0.64	5.16 ± 2.22
4	45/36	−4.20	−4.82	14.29	10.40

## Discussion

MRCPs have been compared between actual movement and motor imagery (Romero et al., 2000; do Nascimento et al., 2006) and analyzed for different psychological status and movement parameters (Shibasaki and Hallett, 2006). This study compared the features of MRCP in healthy subjects with those in ALS patients. Statistical analysis showed that there was no difference between PN of healthy people and PN of ALS patients during both fast movement imagery and slow movement imagery. BP reflects the movement preparation and planning which depend on sensory and cognitive processing. PN of BP could reflect movement preparation to some extent, therefore obtaining no statistically significant difference between the healthy PN and ALS PN could indicate that ALS patients have largely intact sensory, emotional, and cognitive processing despite of extensive motor system degeneration. However, the RR was faster for the healthy than for the ALS individuals during both fast and slow movement. The RR reflects post-movement related brain activity. The weaker RR in ALS patients might reflect impairment of motor output pathway. In ALS patients in Table 2, patient 1, 2, and 3 had weak RR, while patient 4 had similar RR as the healthy. Patient 4 was in the very early stage of ALS and had normal motor abilities except for a slight weakness of the right index finger, while the other 3 patients were severely disabled. Therefore, the RR might also reflect the degree of motor degeneration. Recently, it has been shown that proprioceptive together with auditory feedback are the only observed open windows to stimulate ALS patients in the CLIS (Ramos Murguialday et al., 2011) and therefore similar analyses on MRCP on passive movements need to be done to explore the potential of using MRCP based BCI in the transition from the LIS to the CLIS in ALS patients.

In this study, ALS patients presented quite different status and different ALS-FRS. However, 3 patients selected for statistical analysis shared one common feature: they all had no movements at right wrist. Since the experimental task was imaging right wrist movements, this feature made these three ALS patients into a homogeneous patient group. Therefore, comparison between the healthy and ALS patients was quite reasonable. The healthy subjects were younger than the patients. The experimental tasks were simple and do not require strength and fine skills related to age. Patient 4 who was 70-years old with normal wrist movements presented quite similar RR as the young and healthy subjects. Singh et al. (1990) examined the age effect on MRCP by comparing young group (mean age = 29.3) and old group (mean age = 67.2). The result indicated that MRCP resulted from voluntary movements were unaffected by normal aging. The number of subjects investigated in the study was small. However, One-sided wilcoxon rank-sum test showed that there were significant differences on Fast RR (*p* = 0.008) and Slow RR (*p* = 0.017) between the healthy and ALS patients. Here, *p*-value for fast RR is 0.008 which is less than 0.01 and *p*-value for slow RR is 0.017 which is slightly more than 0.01. Those small *p*-values showed strong evidence against the null hypothesis in favor of the alternative (Anderson et al., 2008). The analysis of MRCP should be examined on larger patient populations to explore the full potential and make strong conclusion in the future. In the study, we chose PN and RR as comparison features. We did not compare the timing of PN between two groups. Because real imagination onset varied greatly among trials even the paradigm provided the cueing timing for initiation, the timing of PN varied among trials. However, the way we extracted RR and PN avoided this timing variation. PN was calculated as lowest value between −1 and 2 s, in which imagination started mostly. RR was calculated as the difference of 1 s after PN with respect to PN. RR and PN were extracted from post-movement potential and believed to reflect characteristic of imagined movement mostly without worrying varied imagination onset.

The comparison between the healthy and the patients might provide some hints to explain movement disorders by means of brain electrical activity. It might pave way to use features of MRCP as a no-invasively diagnostic tool for motor impairment. For BCI application in communication and control, intensive research has been devoted to discriminate simple motor imagery of different limbs to increase the degree of freedom of BCI (Blankertz et al., 2003; McFarland and Wolpaw, 2005; Pfurtscheller et al., 2006). Our research has shown that MRCPs modulated by movement parameters from one limb could be classified on both healthy subjects and ALS patients (Farina et al., 2007; do Nascimento and Farina, 2008; Gu et al., 2009c). Therefore, features of MRCP such as RR could serve as an alternative or supplementary control signal for BCI. For successful motor rehabilitation, induced plasticity must be identified. MRCP spatial and temporal characteristics are suitable to be used to identify induced brain plasticity in patients. Relevant features of MRCP such as RR in parallel with motor control performance could be used to assess motor recovery and decline.

### Conflict of interest statement

The authors declare that the research was conducted in the absence of any commercial or financial relationships that could be construed as a potential conflict of interest.
